# Supplementary material for: A revised model of TRAIL‐R2 DISC assembly explains how FLIP(L) can inhibit or promote apoptosis
Source: EMBO Rep. 2020 Feb 3;21(3):e49254. doi: 10.15252/embr.201949254 (PMC7054686; doi:10.15252/embr.201949254)
Supplement: Supplementary file 1 — Expanded View Figures PDF [file EMBR-21-e49254-s001.pdf]

Expanded View Figures

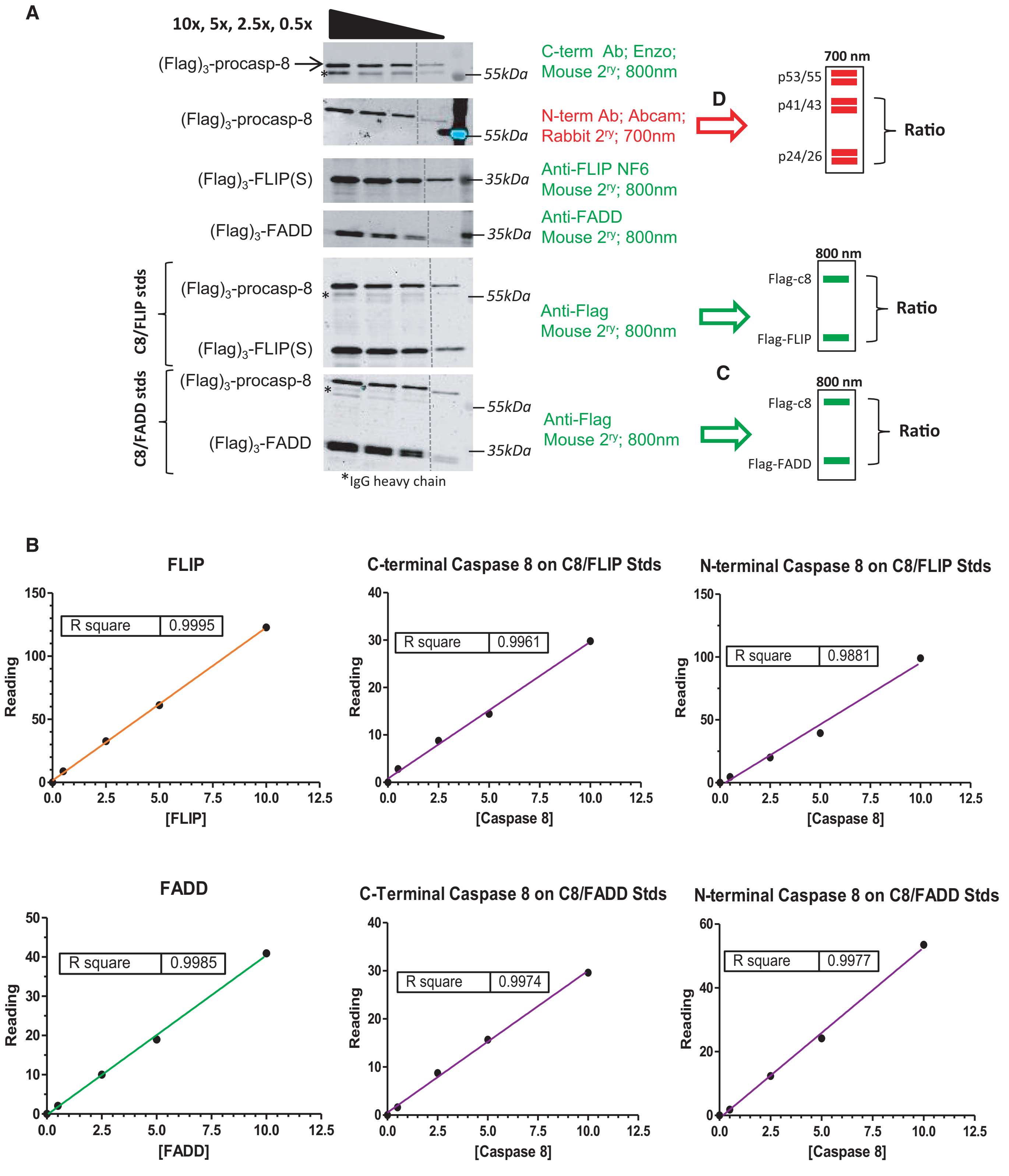

Figure EV1.

© 2020 The Authors

Figure EV1. Quantitative DISC IP methodology.

A Western blot analysis of Flag-tagged-caspase-8/FLIP and Flag-tagged-caspase-8/FADD standards using protein-specific and anti-Flag primary antibodies and LiCOR mouse (800 nm, green) and rabbit (700 nm, red) secondary antibodies.
B In the green channel, standard curves for each exogenous protein were used to calculate the abundance of each protein-specific band in DISC IPs (see Figs 1 and 2, and EV3 and EV4).
C A correction was made to account for any small differences between the procaspase-8 and FLIP (C8/FLIP standards) and procaspase-8 and FADD (C8/FADD standards) bands as detected using the anti-Flag antibody.
D In the red channel, the standard curves were used to calculate the ratio of p41/43-caspase-8 to p24/26-caspase-8; the amount of p24/26-caspase-8 was then calculated from the amount of p41/43-caspase-8 detected in the green channel.

Source data are available online for this figure.

Figure EV2. Baseline expression of TRAIL-R2 interactome and sensitivity to TRAIL-R2 agonists.

A, B Western blot analysis of basal levels of the TRAIL-R2 DISC components FLIP, procaspase 8, FADD and TRAIL-R2 in A549, HCT116 and DU145 cells. Caspase activity (6 h) and cell death induction (24 h) in A549, HCT116 and DU145 cells treated with (B) IZ-TRAIL.
C, D anti-TRAIL-R2 (AMG655)-coated magnetic beads; and (D) MEDI3039. Specific apoptosis was calculated as treatment-induced apoptosis minus background apoptosis in control untreated cells.
E Relative levels of FADD, total caspase-8 and total FLIP pulled down in the TRAIL-R2 DISC IPs presented in Fig 1.

 © 2020 The Authors

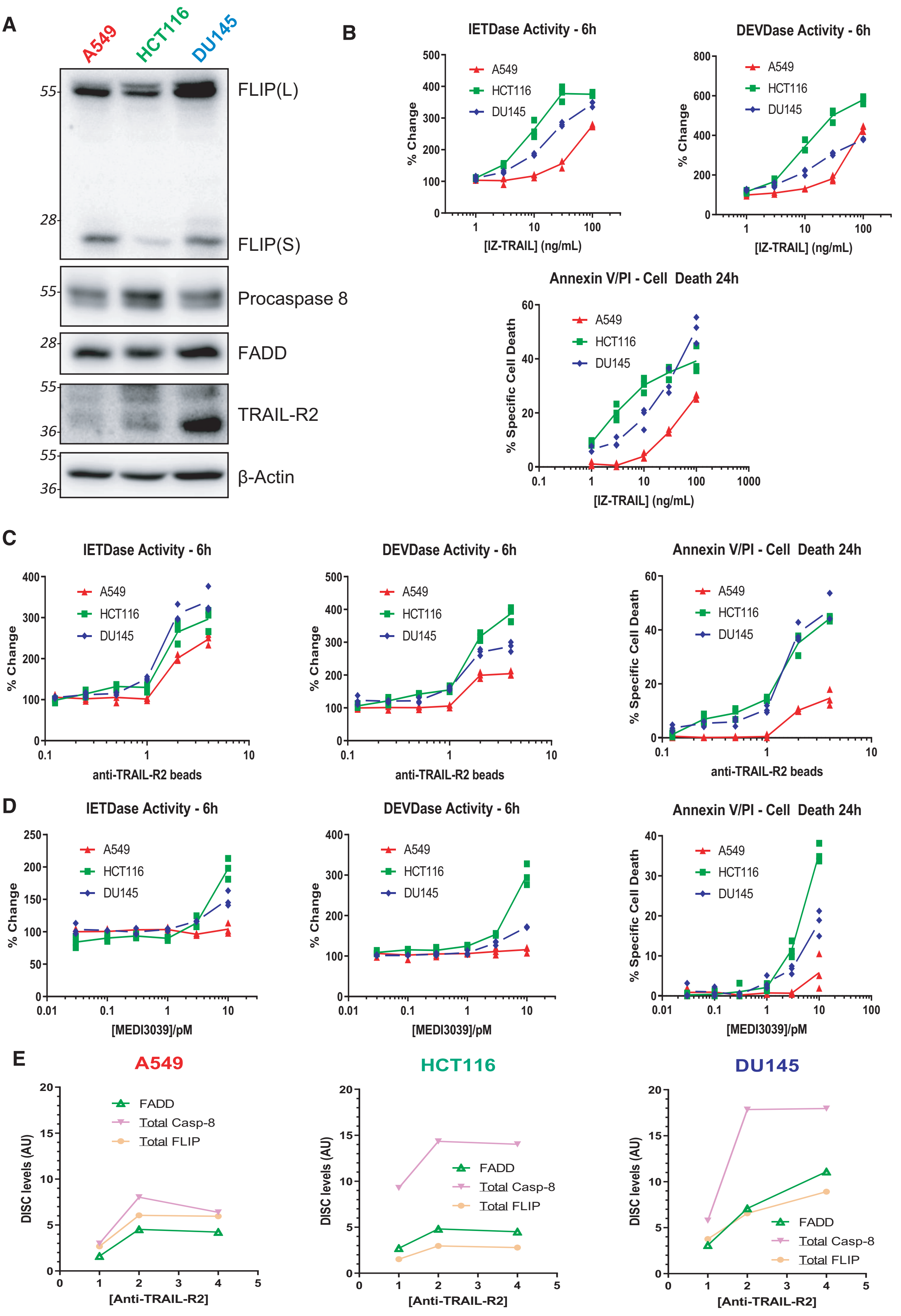
A
A549
HCT116
DU145
55
28
FLIP(L)
FLIP(S)
55
Procaspase 8
28
FADD
55
36
TRAIL-R2
55
36
β-Actin
B
IETDase Activity - 6h
DEVDase Activity - 6h
A549
HCT116
DU145
% Change
[IZ-TRAIL] (ng/mL)
Annexin V/PI - Cell Death 24h
% Specific Cell Death
C
IETDase Activity - 6h
DEVDase Activity - 6h
Annexin V/PI - Cell Death 24h
anti-TRAIL-R2 beads
D
IETDase Activity - 6h
DEVDase Activity - 6h
Annexin V/PI - Cell Death 24h
[MEDI3039]/pM
E
A549
HCT116
DU145
FADD
Total Casp-8
Total FLIP
DISC levels (AU)
[Anti-TRAIL-R2]

Figure EV2.

Figure EV3. Impact of pan-caspase inhibition on DISC stoichiometry.

A Western blot analysis of FLIP, FADD and procaspase-8 recruitment to the TRAIL-R2 DISC in A549, HCT116 and DU145 cells pre-treated with 20 μM zVAD-fmk or DMSO followed by incubation with AMG655-conjugated magnetic beads for 90 min.

B Caspase-8 (IETDase) activity assay of the soluble unbound fraction from panel (A).

C–G Quantification of FADD, caspase-8, FLIP and their respective cleavage fragments from panel (A). TRAIL-R2 DISC IP ratios calculated from several independent experiments for (D) caspase-8:FADD; (E) caspase-8:FLIP; (F) FLIP:FADD; and (G) {Caspase-8+FLIP}:FADD.

Source data are available online for this figure.

 © 2020 The Authors

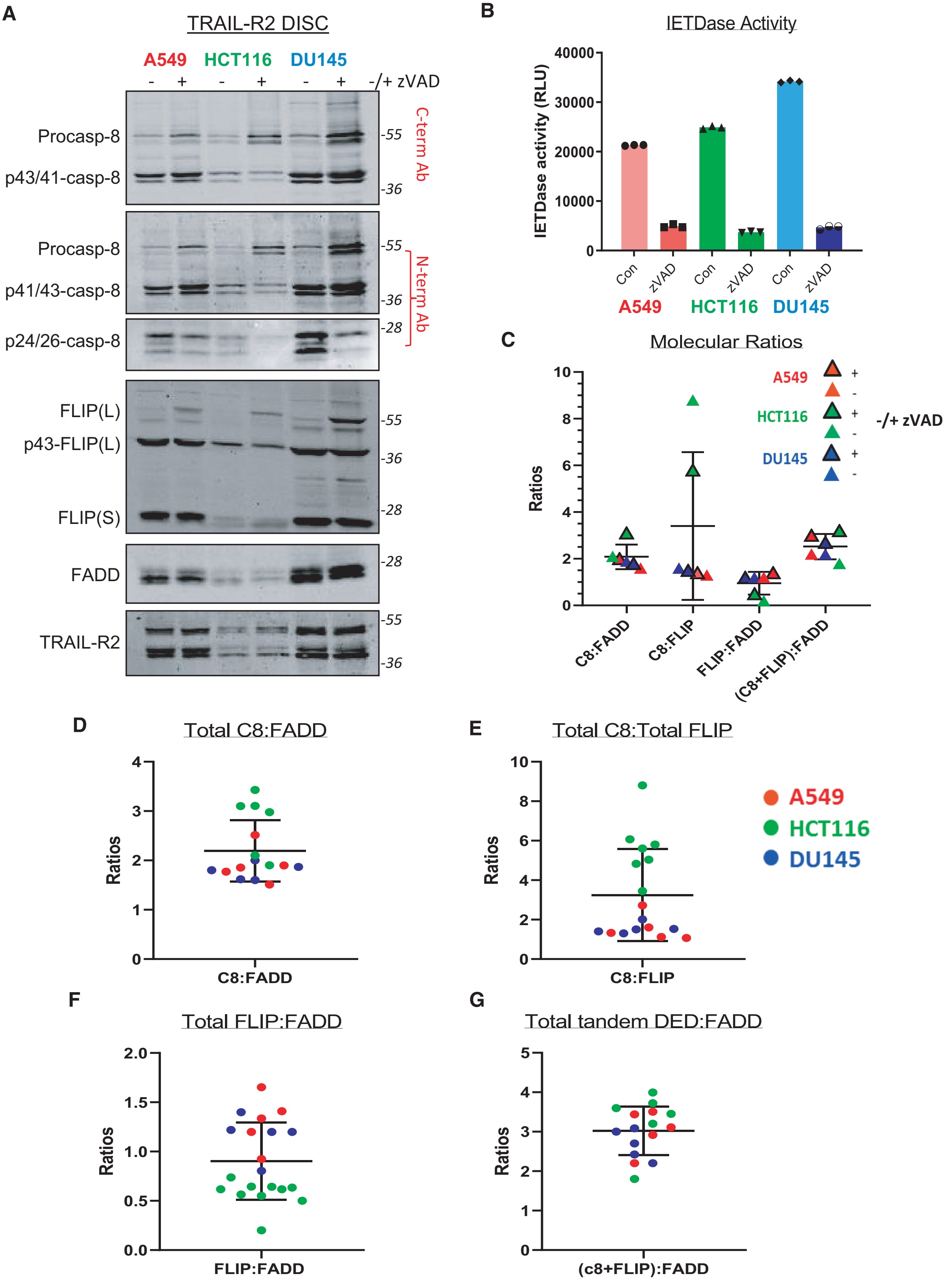
A
TRAIL-R2 DISC
A549 HCT116 DU145
- + - + - + -/+ zVAD
Procasp-8
p43/41-casp-8
C-term Ab
Procasp-8
p41/43-casp-8
p24/26-casp-8
N-term Ab
FLIP(L)
p43-FLIP(L)
FLIP(S)
FADD
TRAIL-R2
-55
-36
-28
B
IETDase Activity
IETDase activity (RLU)
40000
30000
20000
10000
0
Con
zVAD
A549
HCT116
DU145
C
Molecular Ratios
Ratios
A549
HCT116
DU145
-/+ zVAD
C8:FADD
C8:FLIP
FLIP:FADD
(C8+FLIP):FADD
D
Total C8:FADD
Ratios
C8:FADD
E
Total C8:Total FLIP
C8:FLIP
F
Total FLIP:FADD
FLIP:FADD
G
Total tandem DED:FADD
(c8+FLIP):FADD

Figure EV3.

Figure EV4. Impact of procaspase-8 downregulation on DISC stoichiometry.

A Western blot analysis of caspase-8, FLIP, FADD and TRAIL-R2 in the unbound soluble fraction from Fig 2A.
B Correlation of levels of caspase-8 cleavage fragments (p43/41 and p26/24) from Fig 2A with caspase-8 activity quantified in Fig 2D.
C Western blot analysis of proteins recruited to the TRAIL-R2 DISC following a 48-h treatment with caspase-8-targeting siRNA (0–30 nM) in HCT116 BAX/BAK null cells.
D Quantification of caspase-8 (p55, p41/43, p24/26), FLIP (FLIP(L), p43-FLIP, FLIP(S)) and FADD at the TRAIL-R2 DISC from panel (C).
E Caspase activity assays from the unbound soluble fraction from panel (C).
F Quantification of FLIP, FADD and caspase-8 at the TRAIL-R2 DISC in panel (C).

Source data are available online for this figure.

© 2020 The Authors

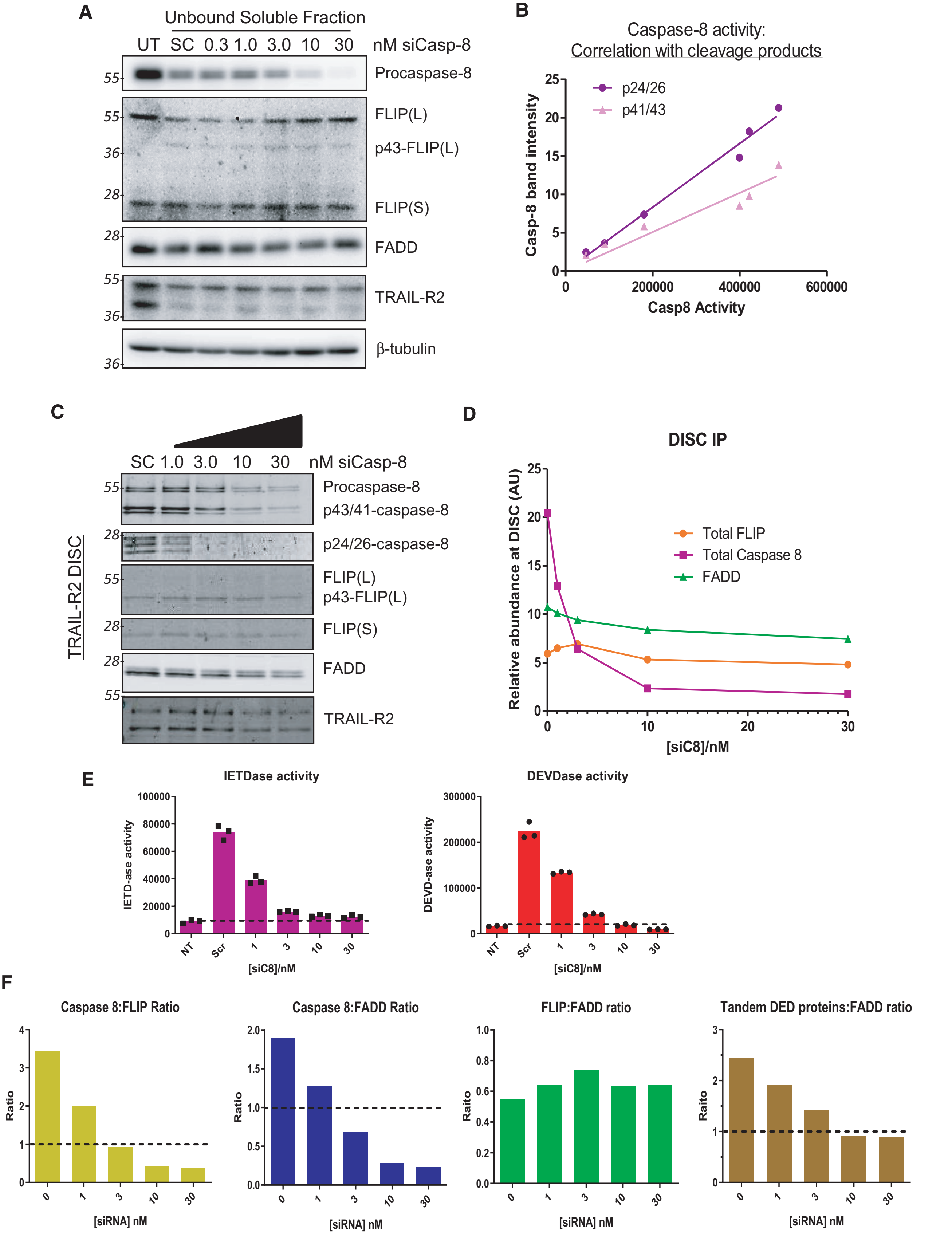
A
Unbound Soluble Fraction
UT SC 0.3 1.0 3.0 10 30 nM siCasp-8
Procaspase-8
FLIP(L)
p43-FLIP(L)
FLIP(S)
FADD
TRAIL-R2
β-tubulin
55
55
36
28
28
55
36
36
B
Caspase-8 activity:
Correlation with cleavage products
p24/26
p41/43
Casp-8 band intensity
Casp8 Activity
C
SC 1.0 3.0 10 30 nM siCasp-8
TRAIL-R2 DISC
Procaspase-8
p43/41-caspase-8
p24/26-caspase-8
FLIP(L)
p43-FLIP(L)
FLIP(S)
FADD
TRAIL-R2
D
DISC IP
Total FLIP
Total Caspase 8
FADD
Relative abundance at DISC (AU)
[siC8]/nM
E
IETDase activity
IETD-ase activity
DEVDase activity
DEVD-ase activity
NT
Scr
[siC8]/nM
F
Caspase 8:FLIP Ratio
Caspase 8:FADD Ratio
FLIP:FADD ratio
Tandem DED proteins:FADD ratio
Ratio
Raito
[siRNA] nM

Figure EV4.

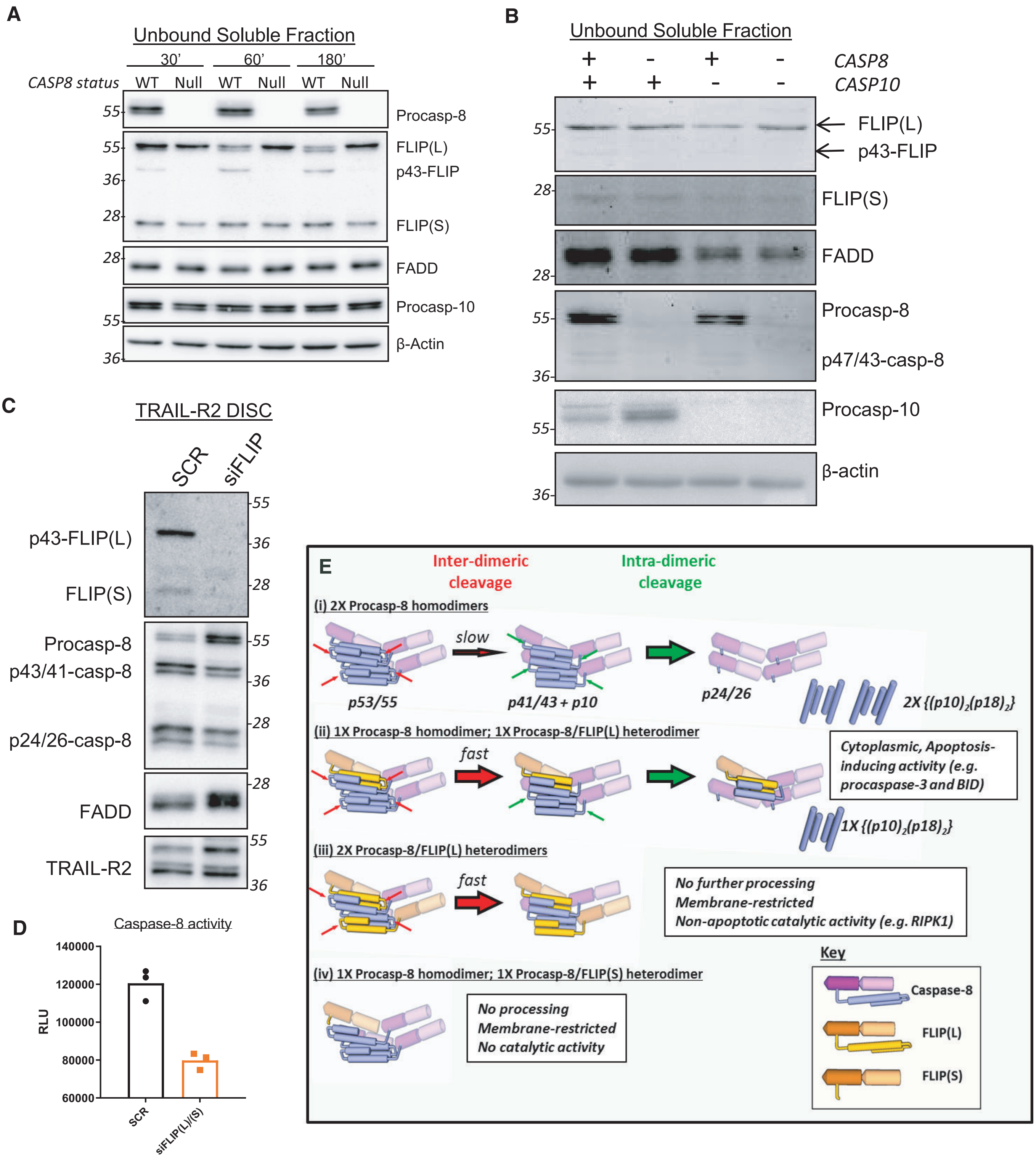

Figure EV5.

 © 2020 The Authors

Figure EV5. Impact of FLIP depletion on DISC stoichiometry and schematic to explain procaspase-8 and FLIP processing events at the DISC.

A Western blot analysis of FLIP, caspase-8, caspase-10 and FADD in the soluble unbound fraction from Fig 3A.
B Western blot analysis of FLIP, caspase-8, caspase-10 and FADD in the soluble unbound fraction from Fig 3B.
C Western blot analysis of a 90-min DISC IP in HCT116 BAX/BAK null cells treated with 100nM Scramble (SCR) or FLIP targeting (siFLIP) siRNA for 24 h.
D Caspase-8 activity assay in the soluble unbound fraction.
E Schematic of the processing of procaspase-8 and FLIP in the various DISC assembly scenarios depicted in the proposed models in Fig 6 and catalytic activities of the products formed.

© 2020 The Authors
