# Supplementary material for: A revised model of TRAIL‐R2 DISC assembly explains how FLIP(L) can inhibit or promote apoptosis
Source: EMBO Rep. 2020 Feb 3;21(3):e49254. doi: 10.15252/embr.201949254 (PMC7054686; doi:10.15252/embr.201949254)
Supplement: Supplementary file 2 — Source Data for Expanded View [file EMBR-21-e49254-s007.zip › Source_Data_for_EV_Figures/Source_Data_for_FigEV1.pptx]

## Slide 1
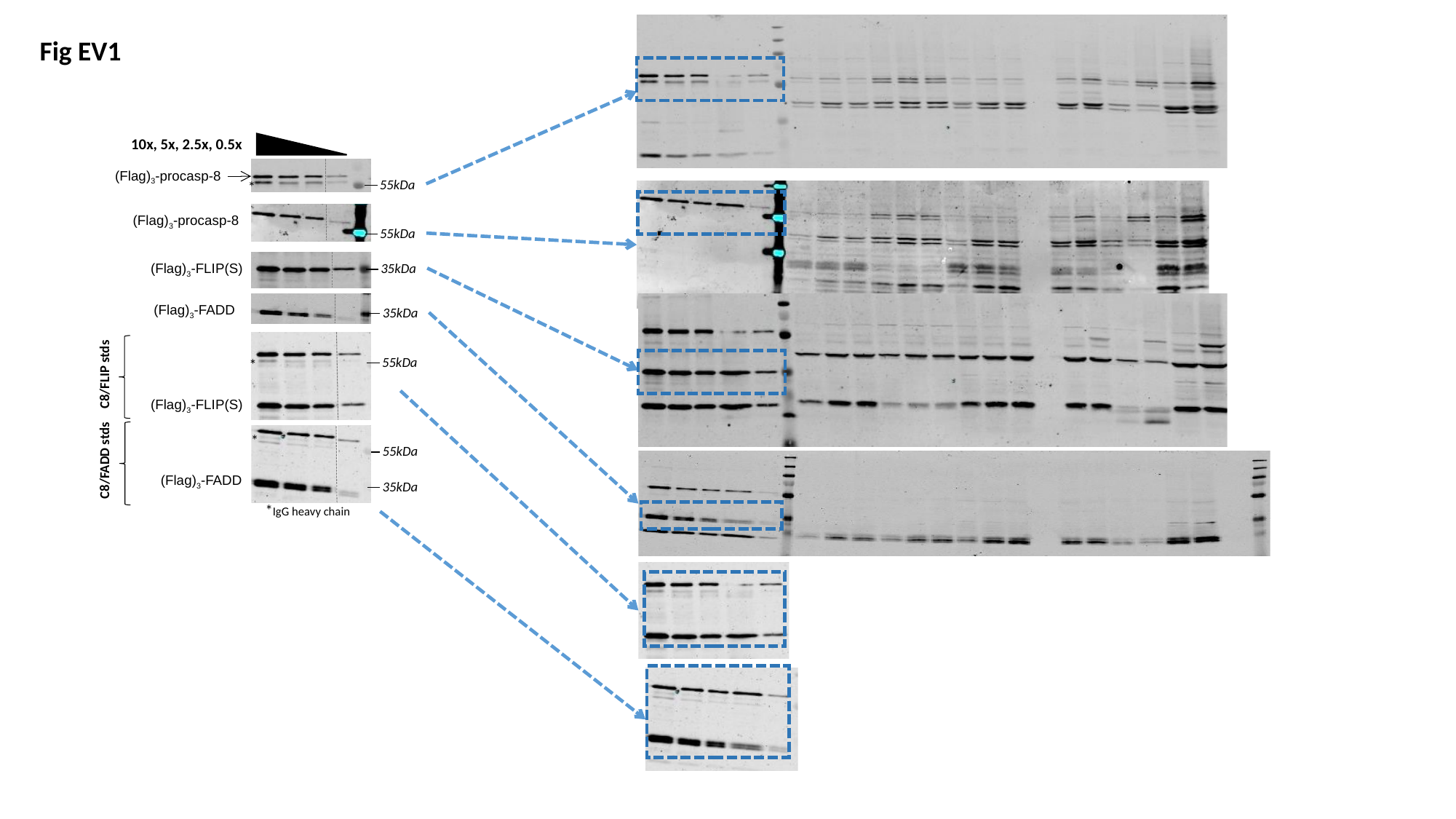

Fig EV1
10x, 5x, 2.5x, 0.5x
55kDa
*
55kDa
35kDa
(Flag)3-FLIP(S)
(Flag)3-FADD
35kDa
55kDa
*
C8/FLIP stds
(Flag)3-FLIP(S)
*
55kDa
C8/FADD stds
(Flag)3-FADD
35kDa
*IgG heavy chain
(Flag)3-procasp-8
(Flag)3-procasp-8
