# Supplementary material for: A revised model of TRAIL‐R2 DISC assembly explains how FLIP(L) can inhibit or promote apoptosis
Source: EMBO Rep. 2020 Feb 3;21(3):e49254. doi: 10.15252/embr.201949254 (PMC7054686; doi:10.15252/embr.201949254)
Supplement: Supplementary file 2 — Source Data for Expanded View [file EMBR-21-e49254-s007.zip › Source_Data_for_EV_Figures/Source_Data_for_FigEV3.pptx]

## Slide 1
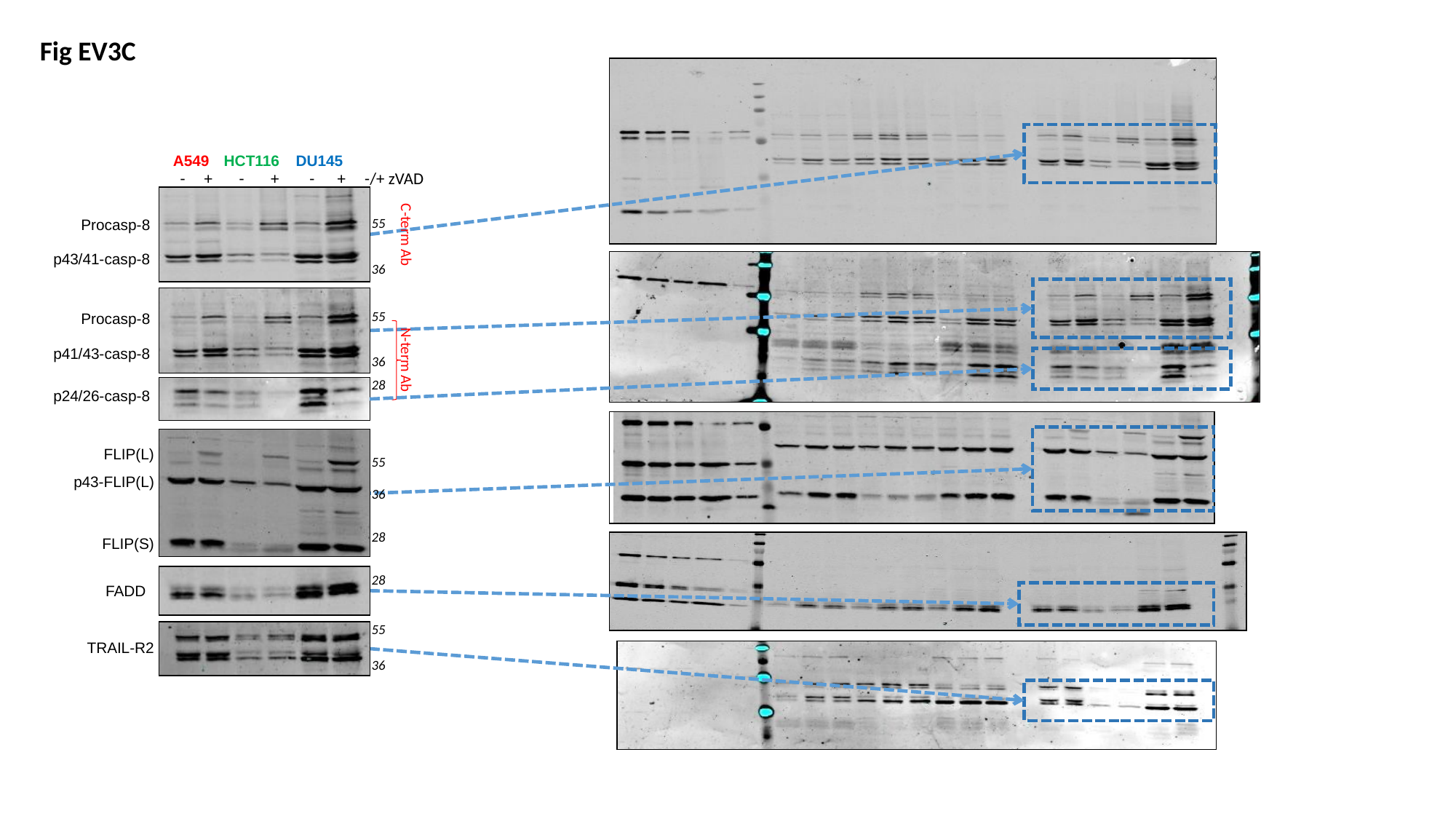

Fig EV3C
A549
HCT116
DU145
- + - + - + -/+ zVAD
Procasp-8
C-term Ab
p43/41-casp-8
Procasp-8
p41/43-casp-8
N-term Ab
p24/26-casp-8
FLIP(L)
p43-FLIP(L)
FLIP(S)
FADD
TRAIL-R2
55
36
55
36
28
55
36
28
28
55
36
