# Supplementary material for: A revised model of TRAIL‐R2 DISC assembly explains how FLIP(L) can inhibit or promote apoptosis
Source: EMBO Rep. 2020 Feb 3;21(3):e49254. doi: 10.15252/embr.201949254 (PMC7054686; doi:10.15252/embr.201949254)
Supplement: Supplementary file 2 — Source Data for Expanded View [file EMBR-21-e49254-s007.zip › Source_Data_for_EV_Figures/Source_Data_for_FigEV4.pptx]

## Slide 1
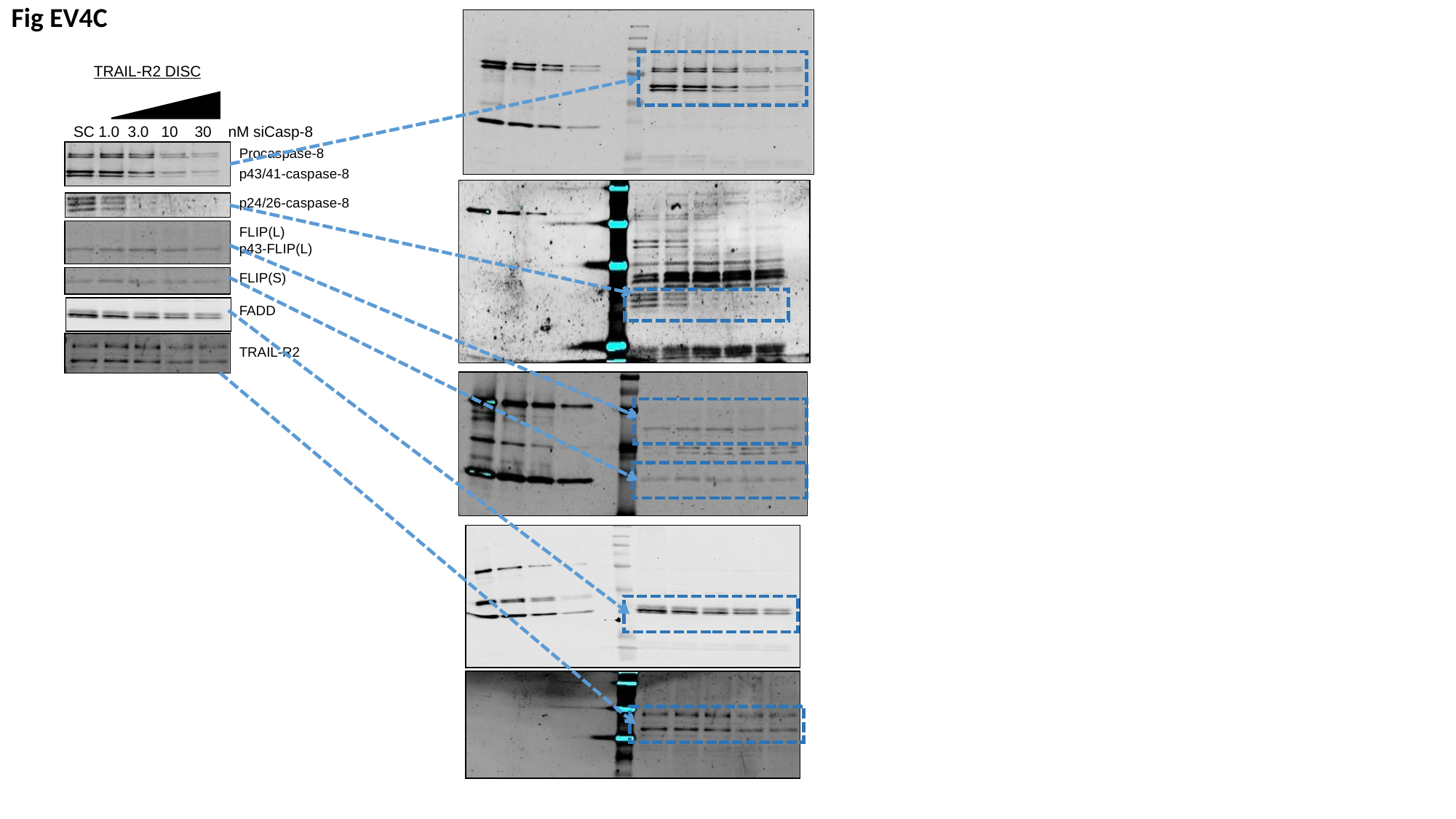

Fig EV4C
TRAIL-R2 DISC
SC 1.0 3.0 10 30 nM siCasp-8
Procaspase-8
p43/41-caspase-8
p24/26-caspase-8
FLIP(L)
p43-FLIP(L)
FLIP(S)
FADD
TRAIL-R2
