# Supplementary material for: A revised model of TRAIL‐R2 DISC assembly explains how FLIP(L) can inhibit or promote apoptosis
Source: EMBO Rep. 2020 Feb 3;21(3):e49254. doi: 10.15252/embr.201949254 (PMC7054686; doi:10.15252/embr.201949254)
Supplement: Supplementary file 4 — Source Data for Figure 1 [file EMBR-21-e49254-s002.pptx]

## Slide 1
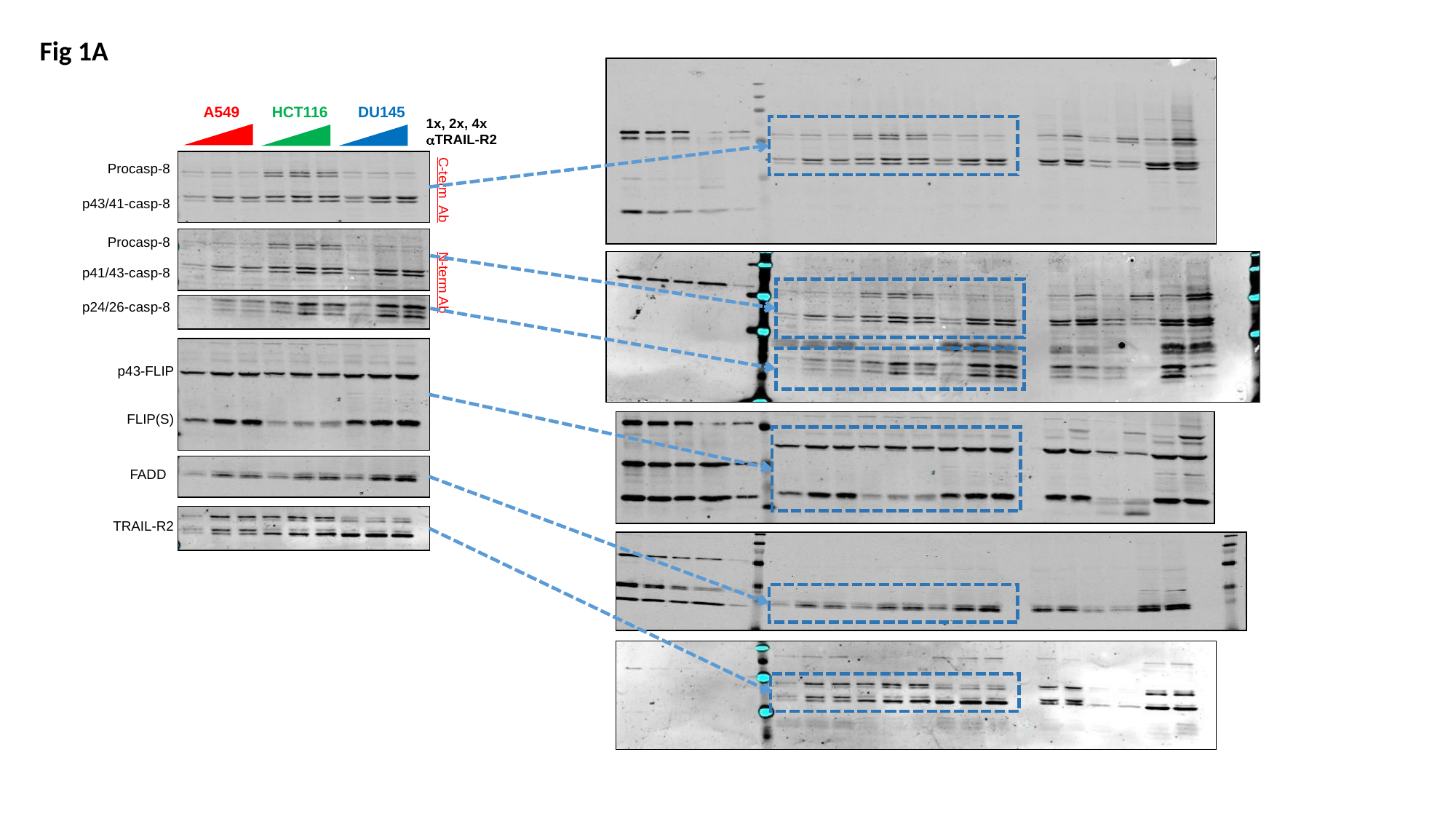

Fig 1A
A549
HCT116
DU145
1x, 2x, 4x
aTRAIL-R2
Procasp-8
C-term Ab
p43/41-casp-8
Procasp-8
p41/43-casp-8
N-term Ab
p24/26-casp-8
p43-FLIP
FLIP(S)
FADD
TRAIL-R2
