# Supplementary material for: A revised model of TRAIL‐R2 DISC assembly explains how FLIP(L) can inhibit or promote apoptosis
Source: EMBO Rep. 2020 Feb 3;21(3):e49254. doi: 10.15252/embr.201949254 (PMC7054686; doi:10.15252/embr.201949254)
Supplement: Supplementary file 5 — Source Data for Figure 2 [file EMBR-21-e49254-s003.pptx]

## Slide 1
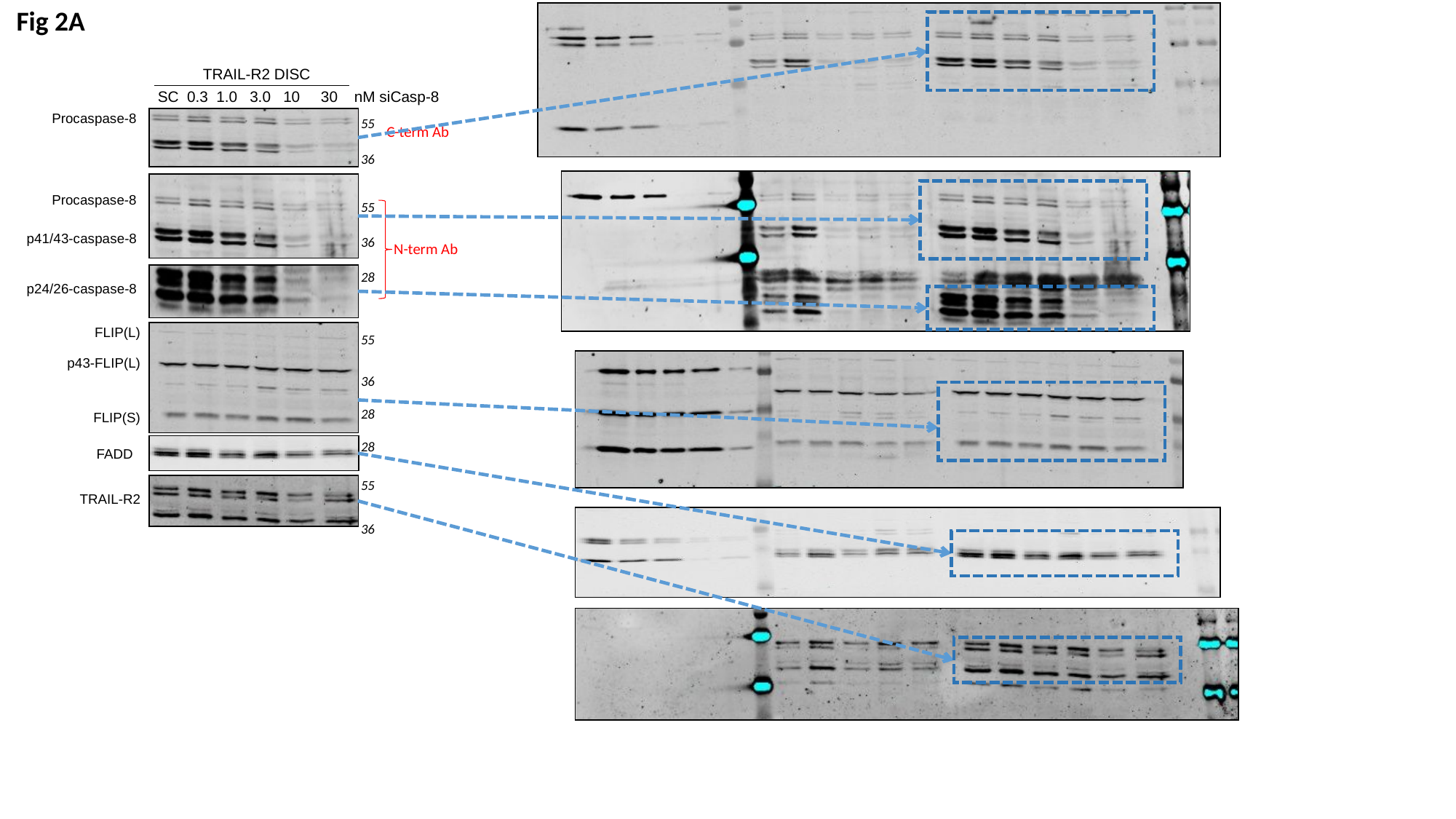

Fig 2A
TRAIL-R2 DISC
SC 0.3 1.0 3.0 10 30 nM siCasp-8
Procaspase-8
55
36
C-term Ab
Procaspase-8
55
36
28
p41/43-caspase-8
N-term Ab
p24/26-caspase-8
FLIP(L)
55
36
28
p43-FLIP(L)
FLIP(S)
28
FADD
55
36
TRAIL-R2

## Slide 2
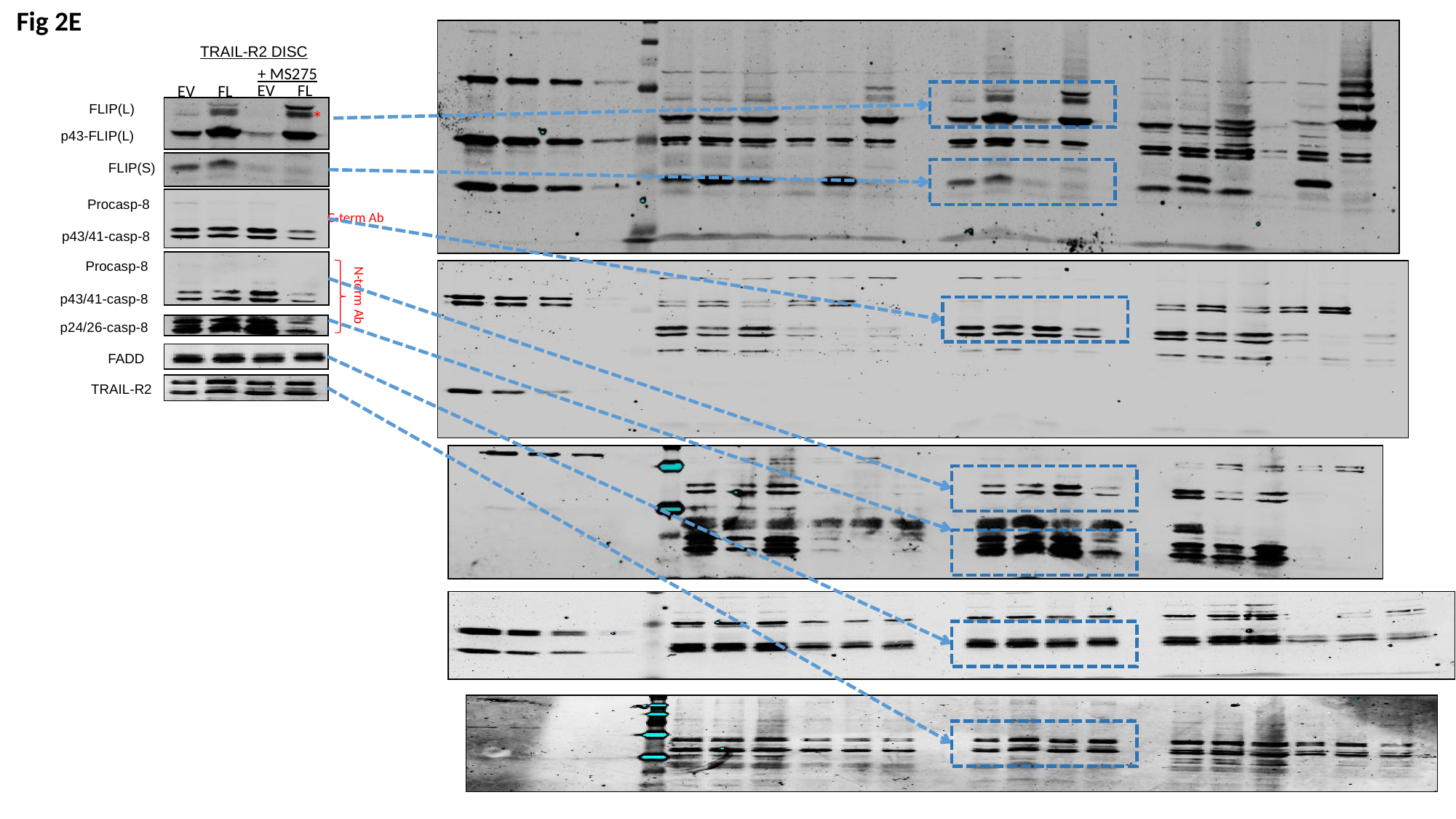

Fig 2E
TRAIL-R2 DISC
+ MS275
 EV FL
EV FL
FLIP(L)
*
p43-FLIP(L)
FLIP(S)
Procasp-8
C-term Ab
p43/41-casp-8
Procasp-8
N-term Ab
p43/41-casp-8
p24/26-casp-8
FADD
TRAIL-R2
