# Supplementary material for: A revised model of TRAIL‐R2 DISC assembly explains how FLIP(L) can inhibit or promote apoptosis
Source: EMBO Rep. 2020 Feb 3;21(3):e49254. doi: 10.15252/embr.201949254 (PMC7054686; doi:10.15252/embr.201949254)
Supplement: Supplementary file 6 — Source Data for Figure 3 [file EMBR-21-e49254-s004.pptx]

## Slide 1
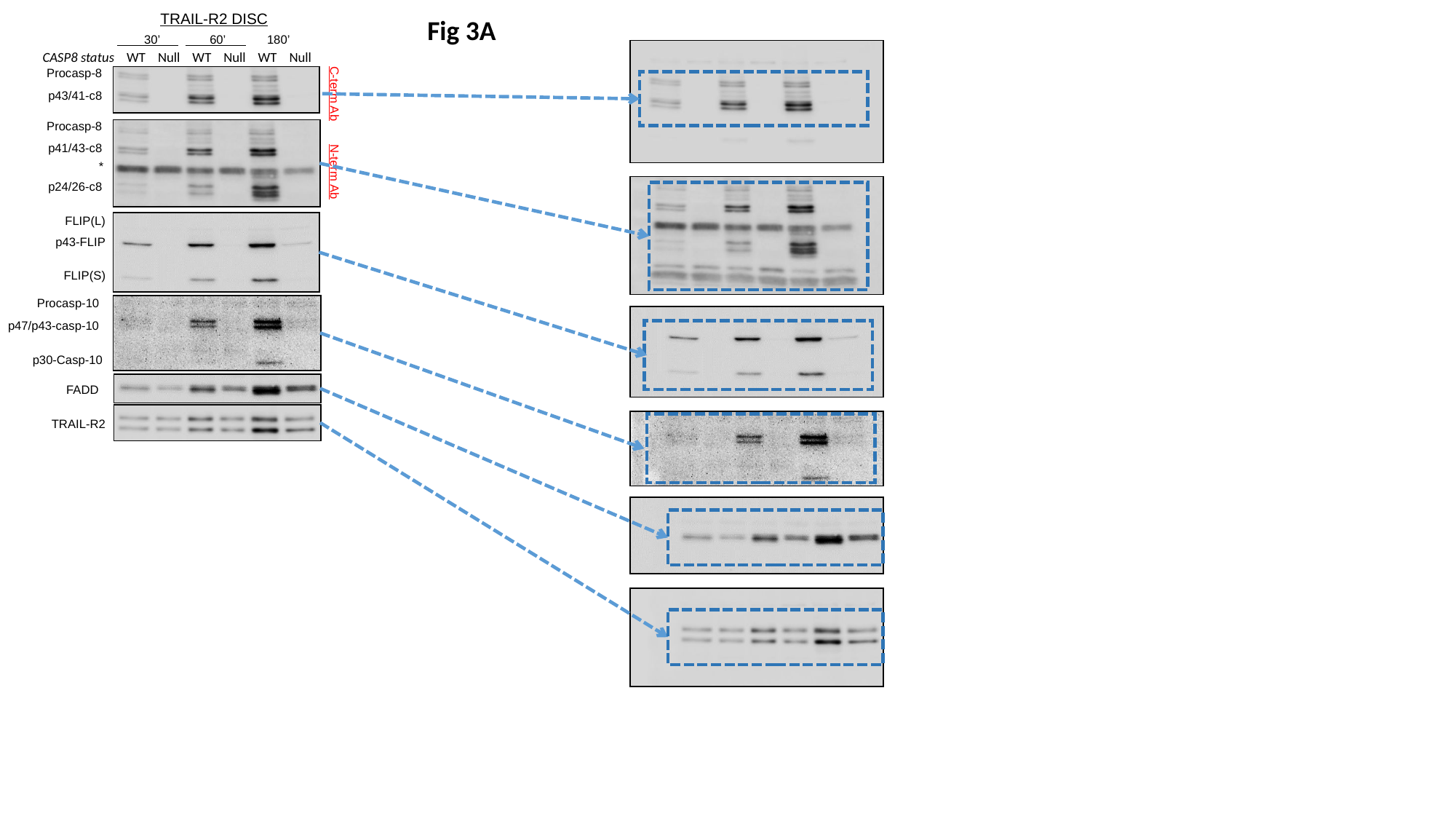

TRAIL-R2 DISC
Fig 3A
30’
60’
180’
CASP8 status WT Null WT Null WT Null
Procasp-8
C-term Ab
p43/41-c8
Procasp-8
p41/43-c8
*
N-term Ab
p24/26-c8
FLIP(L)
p43-FLIP
FLIP(S)
Procasp-10
p47/p43-casp-10
p30-Casp-10
FADD
TRAIL-R2

## Slide 2
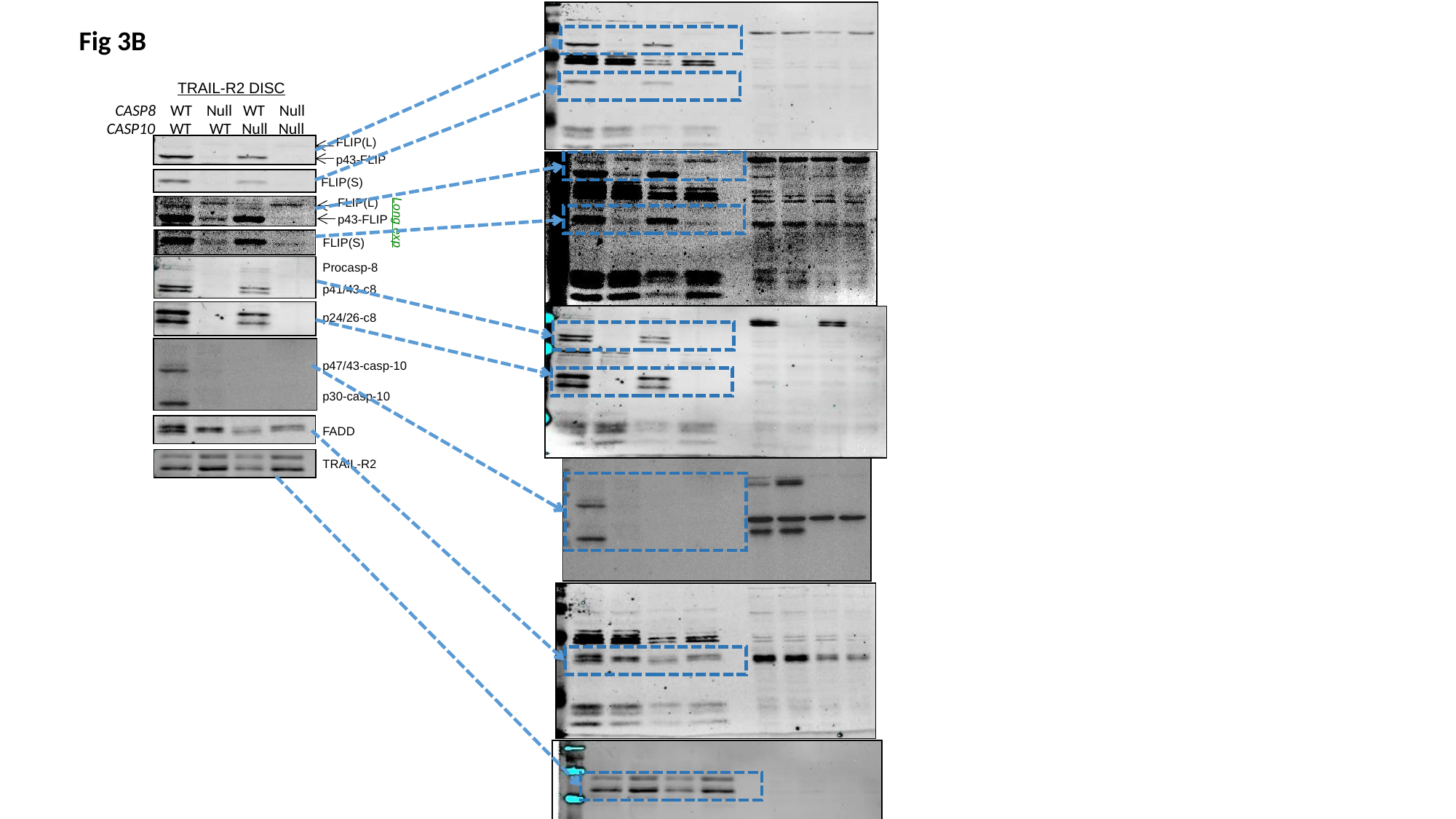

Fig 3B
TRAIL-R2 DISC
CASP8 WT Null WT Null
CASP10 WT WT Null Null
FLIP(L)
p43-FLIP
FLIP(S)
FLIP(L)
p43-FLIP
Long exp
FLIP(S)
Procasp-8
p41/43-c8
p24/26-c8
p47/43-casp-10
p30-casp-10
FADD
TRAIL-R2
