# Supplementary material for: A revised model of TRAIL‐R2 DISC assembly explains how FLIP(L) can inhibit or promote apoptosis
Source: EMBO Rep. 2020 Feb 3;21(3):e49254. doi: 10.15252/embr.201949254 (PMC7054686; doi:10.15252/embr.201949254)
Supplement: Supplementary file 7 — Source Data for Figure 4 [file EMBR-21-e49254-s005.pptx]

## Slide 1
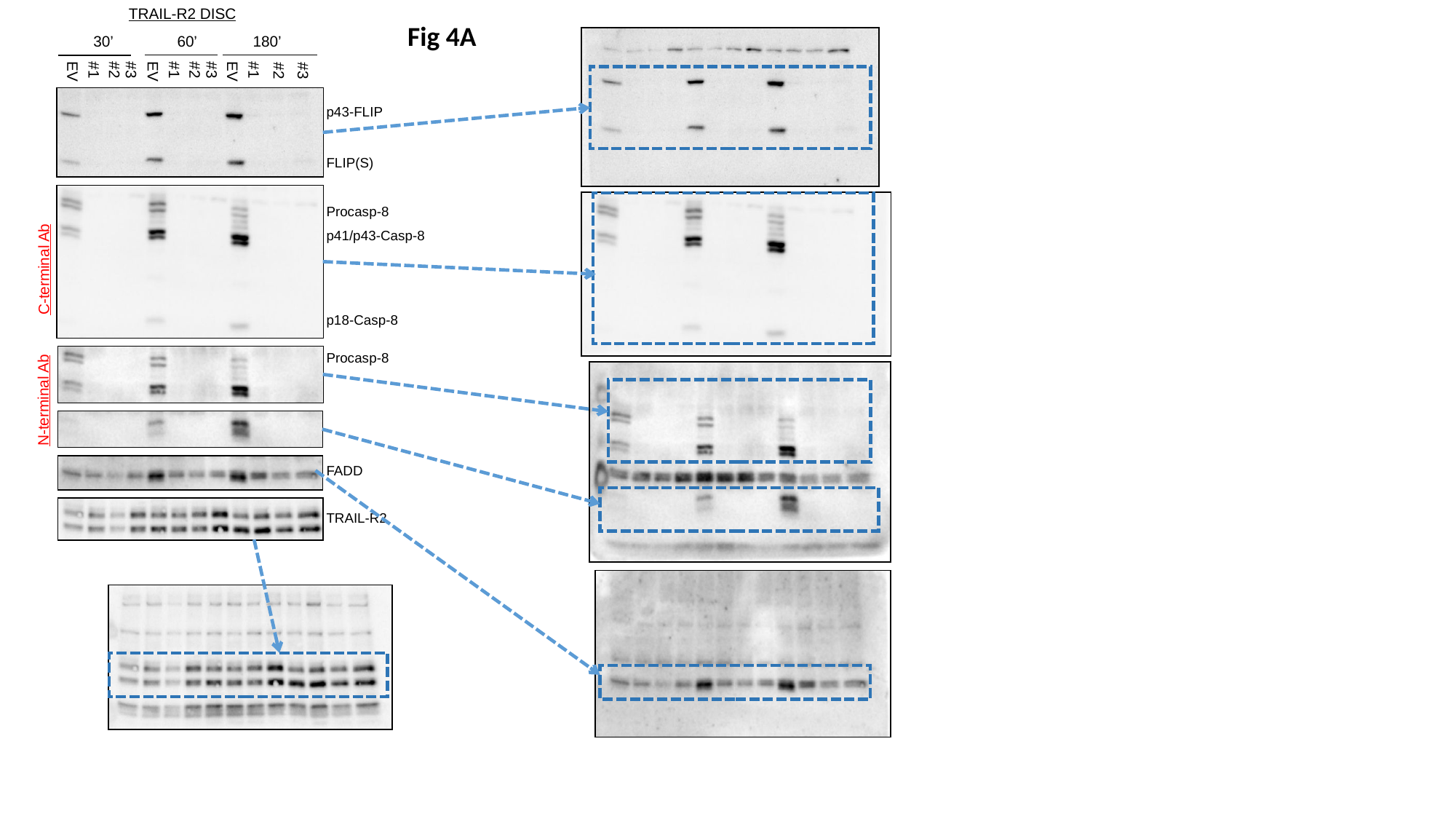

TRAIL-R2 DISC
Fig 4A
30’
60’
180’
EV
#1
#2
#3
EV
#1
#2
#3
EV
#1
#3
#2
p43-FLIP
FLIP(S)
Procasp-8
p41/p43-Casp-8
C-terminal Ab
p18-Casp-8
Procasp-8
N-terminal Ab
FADD
TRAIL-R2

## Slide 2
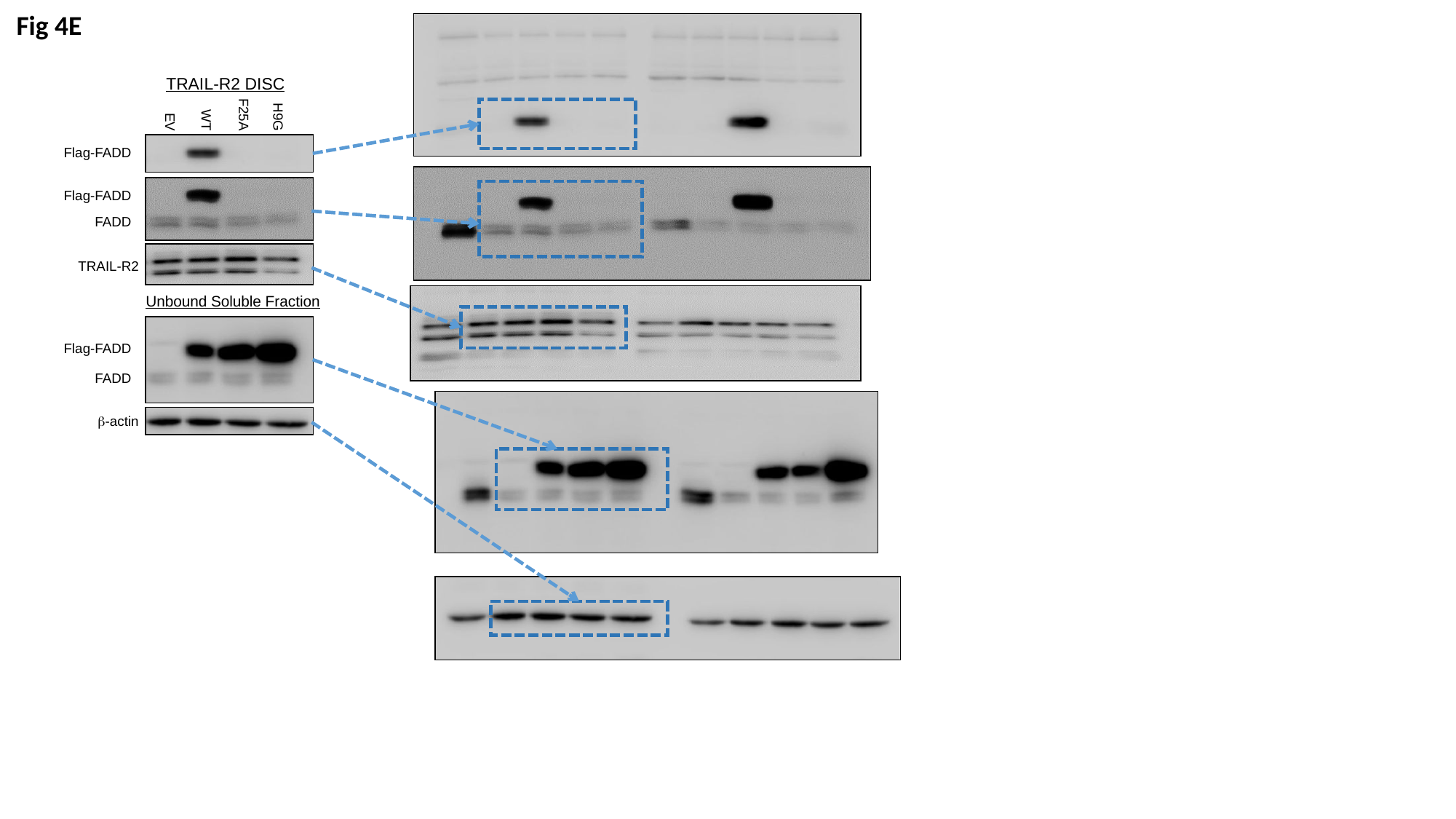

Fig 4E
WT
EV
F25A
H9G
Flag-FADD
Flag-FADD
FADD
TRAIL-R2
Flag-FADD
FADD
b-actin
TRAIL-R2 DISC
Unbound Soluble Fraction
