# Supplementary figures and images for: A revised model of TRAIL‐R2 DISC assembly explains how FLIP(L) can inhibit or promote apoptosis
Source: EMBO Rep. 2020 Feb 3;21(3):e49254. doi: 10.15252/embr.201949254 (PMC7054686; doi:10.15252/embr.201949254)

## Slide 1
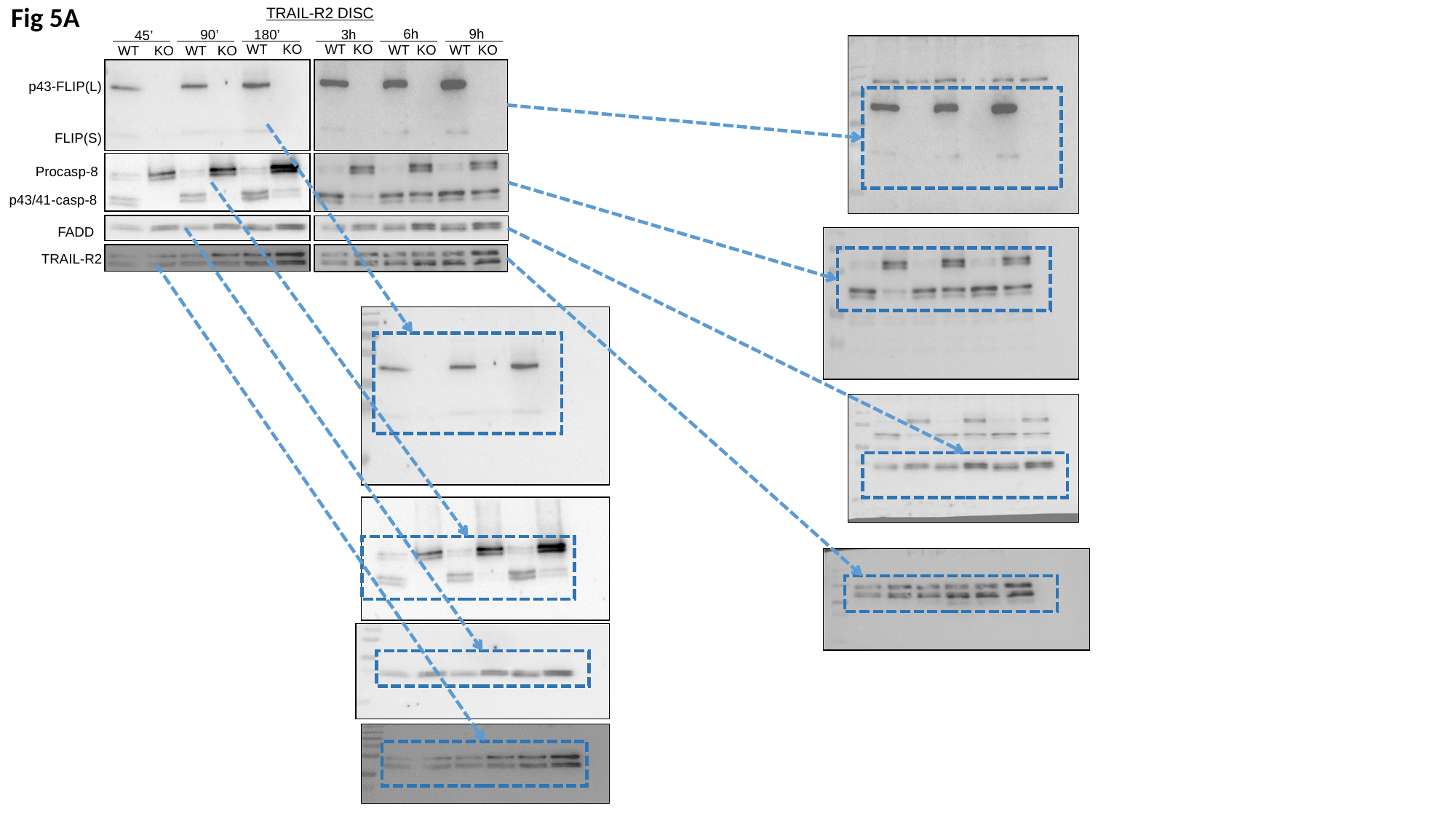

Fig 5A
TRAIL-R2 DISC
6h
9h
3h
90’
180’
45’
WT KO
 WT KO
WT KO
WT KO
WT KO
WT KO
p43-FLIP(L)
FLIP(S)
Procasp-8
p43/41-casp-8
FADD
TRAIL-R2

Supplement: Supplementary file 8 — Source Data for Figure 5 [file EMBR-21-e49254-s006.pptx]
